# PhysAstro-Pose: Physics-Inspired Semi-Supervised Human Pose Estimation in Microgravity Environments

**DOI:** 10.3390/s26113406

**Published:** 2026-05-27

**Authors:** Youhui Cui, Zhang Zhang, Liang Chang

**Affiliations:** 1 Hangzhou Institute for Advanced Study, University of Chinese Academy of Sciences, Hangzhou 310024, China; cuiyouhui24@mails.ucas.ac.cn; 2 Innovation Academy for Microsatellites of Chinese Academy of Sciences, Shanghai 201304, China; zhangz@microsate.com

**Keywords:** human pose estimation, microgravity environment, semi-supervised learning, pseudo-label refinement

## Abstract

Human pose estimation in orbit is critical for astronaut health monitoring, task assistance, and intelligent human–robot interaction aboard space stations. However, in microgravity, human poses exhibit arbitrary orientations and are often affected by severe occlusion and complex background interference, while the scarcity of annotated in-orbit data makes it difficult to directly transfer models trained on ground-based datasets. Existing semi-supervised methods also lack explicit constraints from human structural topology and pose-related physical priors, which often leads to unreasonable pseudo-labels and limits performance gains. To address these issues, we propose a physics-inspired semi-supervised pose estimation framework for microgravity scenarios. Specifically, a Canonical Orientation Constraint is introduced to alleviate orientation ambiguity; a Structure-aware Pseudo-Label Refinement module is designed to improve pseudo-label quality; and an Uncertainty-guided Rotational Consistency Framework is proposed to adaptively weight consistency learning under multi-view rotation augmentation. Within a Mean Teacher architecture, the proposed method jointly optimizes the supervised loss, orientation constraint, pseudo-label refinement, and rotational consistency objectives. Experiments on the Astro-Pose dataset show that the proposed method consistently outperforms both fully supervised and semi-supervised baselines under various extreme poses and occlusion conditions, improving AP from 47.6 to 55.6 and AR from 52.4 to 60.1, demonstrating its potential for space-station visual monitoring.

## 1. Introduction

With the continued advancement of China’s manned space program, the space station has entered a normalized stage characterized by long-term operation and the parallel execution of multiple tasks, leading to a marked increase in the complexity and continuity of astronauts’ on-orbit activities. Operations inside the space station involve not only cabin maintenance, scientific experiments, equipment management, and emergency handling, but also a wide range of activities such as daily exercise and science outreach demonstrations. Under microgravity conditions, changes [1] in postural control strategies, movement patterns, and musculoskeletal loading make conventional assessment approaches based on self-reporting or ground observation inadequate for fine-grained quantification of astronauts’ physiological and behavioral states. In this context, reliable in-cabin astronaut pose estimation is not merely a visual perception task, but an enabling technique for fine-grained activity understanding, health and safety monitoring, task assistance, and intelligent human–machine interaction. Compared with coarse action recognition, pose estimation provides joint-level motion information and can therefore support more detailed analysis of complex astronaut activities and abnormal postural states. Therefore, accurate and non-invasive in-orbit astronaut pose estimation is of substantial importance, as it provides fine-grained joint-level cues for astronaut health management, task assistance, human–robot collaboration, and the optimization of ground-based training strategies.

**Figure 1:**
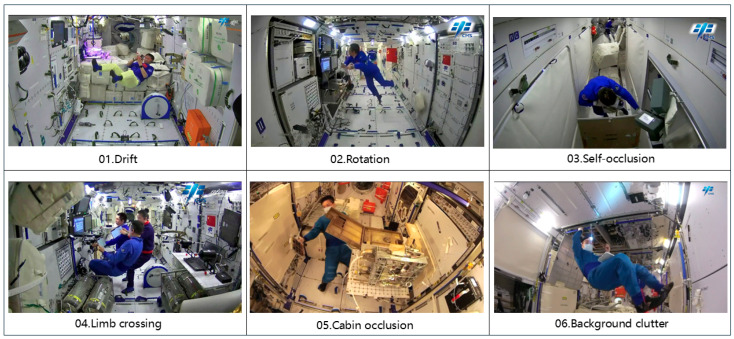
Illustration of the typical challenges in monocular human pose estimation under microgravity. Unlike in terrestrial scenes, astronauts inside a space station often exhibit inverted floating postures, arbitrary body rotations, severe occlusion, and complex background interference.

Vision-based 2D pose estimation provides a feasible solution for on-orbit human pose analysis [2]. However, directly transferring general-purpose models trained on terrestrial data to microgravity environments faces a series of fundamental challenges. One major issue lies in the biomechanical deviations induced by microgravity. Under weightless conditions, astronauts often exhibit a distinctive relaxed posture that differs substantially from the upright or seated poses commonly observed in standard benchmark datasets. In addition, without gravitational constraints, astronauts may move through the space station in unconventional body configurations, such as floating or rotating in arbitrary orientations, often accompanied by severe self-occlusion and inter-person occlusion, as illustrated in Figure 1.

Beyond changes in visual appearance, orientation variations in microgravity introduce a significant distribution shift from terrestrial training data. In ground-based datasets, the human body is usually captured under gravity-aligned conditions, where the head, torso, and limbs follow relatively stable spatial distributions. Consequently, conventional pose estimation models tend to learn an implicit upright-body prior. In contrast, astronauts in microgravity may appear upside down, sideways, or arbitrarily rotated. Such unpredictable orientations weaken this implicit prior and may lead to keypoint localization errors, limb confusion, and structurally inconsistent predictions, especially under self-occlusion, equipment occlusion, and cluttered cabin backgrounds. However, mainstream 2D pose estimation methods, represented by OpenPose [3] and ViTPose [4], are predominantly trained and benchmarked on terrestrial human pose datasets. Given the domain shift between gravity-aligned terrestrial images and orientation-variant on-orbit data, directly applying these models to microgravity environments may reduce their robustness to atypical body configurations and increase the risk of erroneous keypoint localization. To address this issue, a related prior study, Astronaut Pose Net, proposed a voxel-based 3D human pose reconstruction framework for pose estimation in space station environments [5]. Although this approach delivers reliable accuracy, it requires a synchronized multi-camera array and incurs considerable computational cost, making it difficult to deploy on resource-constrained onboard embedded systems. Another critical bottleneck lies in the lack of publicly available, high-quality, annotated on-orbit image datasets. Owing to privacy and security requirements, as well as the specialized nature of this research domain, only a limited portion of real space station cabin imagery is publicly accessible. Moreover, collecting and annotating large-scale real on-orbit image data remains highly time-consuming, labor-intensive, and costly. Although existing studies have attempted to alleviate this issue by constructing synthetic datasets of virtual astronauts [6], a substantial domain gap remains between rendered synthetic data and real cabin imagery, which continues to pose a non-negligible obstacle to model training. Therefore, developing data-efficient algorithmic models is of considerable importance, as it enables effective use of limited annotated data while leveraging semi-supervised learning to fully exploit the potential value of large amounts of unlabeled on-orbit images.

Despite this, directly applying existing semi-supervised pose estimation methods to microgravity environments still involves several challenges. First, because astronauts may float in arbitrary orientations inside the cabin [7], human poses in images often undergo drastic rotational variations, making it difficult for models based on standard convolutional features to maintain stable feature representations [8]. More fundamentally, this difficulty arises from the mismatch between gravity-aligned pose priors learned from terrestrial data and orientation-free pose distributions in microgravity, which may disrupt keypoint spatial relationships and weaken heatmap-based localization reliability. Second, mainstream 2D pose estimation methods typically adopt heatmap-based keypoint prediction, which relies primarily on local pixel-level features but lacks explicit modeling of human skeletal topology. As a result, when visual cues become ambiguous or occlusion occurs, these methods are prone to producing predictions that violate human body structural constraints [9,10,11]. Moreover, in semi-supervised learning, unlabeled data are typically incorporated into training through pseudo labels [12], which often contain substantial noise in complex on-orbit scenarios, further compromising the stability and accuracy of model training. In particular, relying solely on local heatmap confidence may be insufficient to assess pseudo-label reliability when large rotations, self-occlusion, equipment-induced occlusion, and cluttered cabin backgrounds occur simultaneously. Therefore, a microgravity-oriented semi-supervised pose estimation framework should explicitly consider orientation ambiguity, skeletal structural consistency, and uncertainty under substantial pose perturbations.

To further clarify the positioning of this study, Table 1 summarizes representative pose estimation methods, their research focuses, and the gaps addressed by the proposed method. Existing studies have achieved notable progress in general 2D human pose estimation, lightweight real-time deployment, semi-supervised learning, and space-related human pose perception. Nevertheless, they do not fully address the combined challenges of scarce in-orbit annotations, arbitrary-orientation astronaut poses, structural pseudo-label noise, and complex cabin backgrounds in real microgravity environments.

Based on the research gaps summarized in Table 1, this paper presents a semi-supervised monocular 2D astronaut pose estimation method for microgravity environments. The proposed method is designed to address scarce in-orbit annotations, arbitrary pose orientations, structural pseudo-label noise, and uncertainty caused by complex rotational perturbations. By integrating a physics-inspired orientation prior, structure-aware pseudo-label refinement, and uncertainty-guided rotational consistency learning, the proposed framework improves pose estimation robustness and accuracy under limited labeled data. The main contributions are summarized as follows:

(1) A Canonical Orientation Constraint (COC) is introduced for microgravity environments. It establishes a unified image-plane orientation reference to alleviate pose direction ambiguity caused by the absence of a gravity-aligned vertical bias under microgravity conditions.
(2) A Structure-aware Pseudo-Label Refinement (S-PLR) module is proposed to reduce structural pseudo-label noise. By explicitly modeling human skeleton topology, the module refines the initial pseudo labels generated by the teacher network and improves their structural reliability.
(3) An Uncertainty-Guided Rotational Consistency Framework (URCF) is developed to improve robustness to complex pose orientations. Specifically, sample-level uncertainty estimation is used to adaptively weight the rotational consistency loss, thereby enhancing the student network’s robustness to large rotational perturbations and unreliable pseudo labels.
(4) Systematic experiments are conducted on COCO and the self-collected Astro-Pose dataset, with Astro-Pose serving as the target microgravity scenario. The results show that the proposed method consistently improves pose estimation accuracy under limited annotation settings and exhibits stronger robustness in challenging scenarios involving complex rotations and occlusions, providing an efficient and practical solution for human pose perception in space station environments.

## 2. Related Works

### 2.1. 2D Human Pose Estimation

2D human pose estimation [22,23,24] aims to localize the positions of human body joints from images and has become a fundamental research topic in computer vision. It has been widely applied to a variety of downstream tasks, including action recognition, human–computer interaction, and robotic perception. With the rapid development of deep learning, existing methods in this field can generally be categorized into two main paradigms: regression-based methods and heatmap-based methods [25].

Early approaches typically formulated human pose estimation as a direct regression problem over keypoint coordinates. For example, methods such as DeepPose employed deep convolutional networks to predict joint locations directly [26]. However, subsequent studies showed that, because human joints are subject to strong spatial structural constraints, direct coordinate regression often struggles to capture rich spatial context effectively. In contrast, heatmap-based representations model each keypoint as a spatial probability distribution, offering a clear advantage in preserving structural information. As a result, heatmap-based methods have gradually become the dominant paradigm in modern human pose estimation [9].

In terms of network architecture, convolutional neural networks (CNNs) remain the most widely adopted backbone for 2D human pose estimation. SimpleBaseline [27] introduced a concise yet effective architecture that generates high-resolution heatmaps by appending deconvolution layers to a ResNet backbone, achieving strong performance while maintaining structural simplicity. Subsequently, HRNet [13] proposed a parallel multi-resolution architecture that preserves high-resolution representations throughout the network and continuously fuses multi-scale features, substantially improving keypoint localization accuracy. More recently, Transformer-based methods have also been increasingly explored for human pose estimation. Among them, ViTPose [4] leverages the global modeling capability of vision Transformers and achieves strong performance on standard benchmarks such as COCO [28].

Although the aforementioned fully supervised methods have achieved remarkable progress on standard benchmarks, they generally rely on large-scale, high-quality annotated data. In practical applications, however, especially in specialized environments where data collection and annotation are costly, this assumption is often difficult to satisfy. Consequently, how to effectively exploit unlabeled data to improve model performance under limited annotation has become an important research direction in recent years.

### 2.2. 2D Semi-Supervised Human Pose Estimation

Semi-supervised learning (SSL) aims to train models using a small amount of labeled data together with a large amount of unlabeled data, thereby reducing annotation cost and improving generalization ability [29]. In the field of human pose estimation, semi-supervised methods have attracted increasing attention, as keypoint annotation is often labor-intensive and time-consuming.

Existing semi-supervised pose estimation methods are typically built upon strategies such as pseudo-labeling [30] and consistency regularization [31]. Among them, the Mean Teacher framework [32] exploits unlabeled data by enforcing prediction consistency between a teacher network and a student network under different perturbations. Subsequent studies extended this idea to human pose estimation by imposing consistency constraints in the heatmap prediction space, thereby improving the model’s ability to learn from unlabeled data [17].

Building on this line of research, further studies have investigated the role of data augmentation in semi-supervised learning. By applying perturbations of different strengths to the input image and encouraging consistent predictions across augmented views, the model can more effectively exploit structural information from unlabeled data [18]. More recently, MultiAugs [19] adopts a weak-to-strong augmentation strategy, where pseudo labels are first generated from weakly augmented images and then used to supervise the training of strongly augmented counterparts. This design improves pseudo-label quality while enhancing model robustness and has shown promising results on multiple benchmark datasets.

However, existing semi-supervised pose estimation methods typically rely on local criteria, such as heatmap response intensity, keypoint confidence, or uncertainty, to assess prediction reliability during pseudo-label selection. While these metrics primarily reflect local prediction certainty, they are insufficient to fully capture the plausibility of the entire human pose in terms of topological structure and anatomical constraints [33]. In complex scenarios, the model may produce pose predictions with high local confidence but unreasonable overall structure. Once introduced into self-training, such erroneous pseudo labels can be repeatedly reinforced, thereby degrading model performance. Therefore, how to effectively incorporate structural information of human poses into the semi-supervised learning process to improve pseudo-label reliability remains an important issue for further investigation.

### 2.3. Vision Tasks in Microgravity Environments

Microgravity environments introduce unprecedented challenges to computer vision tasks, with the core difficulty arising from the absence of a gravity prior [34]. Unlike in terrestrial scenes, astronauts inside a space station exhibit unconstrained omnidirectional pose distributions, causing conventional models trained under an upright-body assumption to suffer substantial performance degradation when encountering inverted or floating poses. In addition, visual clutter caused by densely arranged onboard equipment, together with complex illumination conditions, further increases the ambiguity of feature extraction [35].

Existing research on in-orbit visual perception remains relatively limited, and early studies have mainly focused on coarse-grained action classification [36], which cannot provide fine-grained joint-level kinematic information. In terms of pose estimation, AstroPose exploits the existing monocular cameras on the space station, together with known exterior structural information, to estimate astronaut pose during extravehicular activities [20], demonstrating the practical feasibility of monocular vision-based solutions in real deployment scenarios. However, this method primarily treats the astronaut as a rigid-body target and estimates the overall pose based on suit feature points and structural priors, making it insufficient for fine-grained human motion analysis. Although $H^{3}$-Astronaut Pose Net achieves high-precision 3D reconstruction [5] through multi-view geometric constraints, its reliance on synchronized multi-camera calibration and high-compute hardware makes it difficult to deploy on resource-constrained in-orbit edge devices. In contrast, monocular 2D pose estimation is more compatible with the practical deployment requirements of space stations due to its lower computational demand and greater hardware flexibility [37]. However, developing a highly robust monocular model remains hindered by severe data and generalization bottlenecks. As highlighted by MicroG-4M [38], there exists a substantial domain shift between gravity-aligned terrestrial data and in-orbit data. Directly transferring existing models often leads to failure in handling unconventional poses. Meanwhile, the high cost of acquiring real in-orbit data and producing fine-grained annotations results in an extreme scarcity of high-quality labeled samples [21].

Taken together, the field is in urgent need of a label-efficient monocular pose estimation method that is robust to large orientation variations. Existing approaches are often limited either by the strong dependence of fully supervised methods on extensive annotations or by the neglect of the unique pose-related constraints induced by microgravity environments. This motivates the present study to explore a semi-supervised learning framework enhanced by physical priors, with the goal of leveraging publicly available unlabeled in-cabin video data to achieve low-cost adaptation to microgravity environments.

## 3. Method

In this section, we present the proposed Physics-Inspired Semi-Supervised Pose Estimation Framework in detail. Section 3.1 formalizes the semi-supervised pose estimation task and outlines the overall training procedure. Section 3.2 introduces the COC, which establishes a unified global orientation reference in the image plane. Section 3.3 describes the S-PLR, which obtains more reliable supervision anchors by structurally refining the initial pseudo labels. Section 3.4 presents the URCF and the overall optimization objective to improve prediction stability and robustness under complex rotational perturbations.

### 3.1. Problem Formulation and Framework Overview

In the semi-supervised 2D pose estimation task, we are given a small labeled dataset $D_{l}=\{\left(x_{i}^{l},y_{i}^{l}\right){\}}_{i=1}^{N_{l}}$ and a large unlabeled dataset $D_{u}=\{\left(x_{j}^{u}\right){\}}_{j=1}^{N_{u}}$, where *x* and *y* denote the input image and the corresponding ground-truth keypoint coordinates, respectively. The goal of semi-supervised learning is to jointly train a pose estimator on both labeled and unlabeled data [18]. The basic loss function can be formulated as:

(1) $$L_{\mathrm{all}}=\frac{1}{N_{l}}\sum_{i=1}^{N_{l}}L_{\sup}\left(x_{i}^{l},y_{i}^{l}\right)+\lambda \frac{1}{N_{u}}\sum_{j=1}^{N_{u}}L_{\mathrm{unsup}}\left(x_{j}^{u},\hat{y_{j}^{u}}\right)$$

where $y_{j}^{u}$ denotes the pseudo-label generated by the teacher model, and λ is the weighting factor for the unsupervised loss.

**Figure 2:**
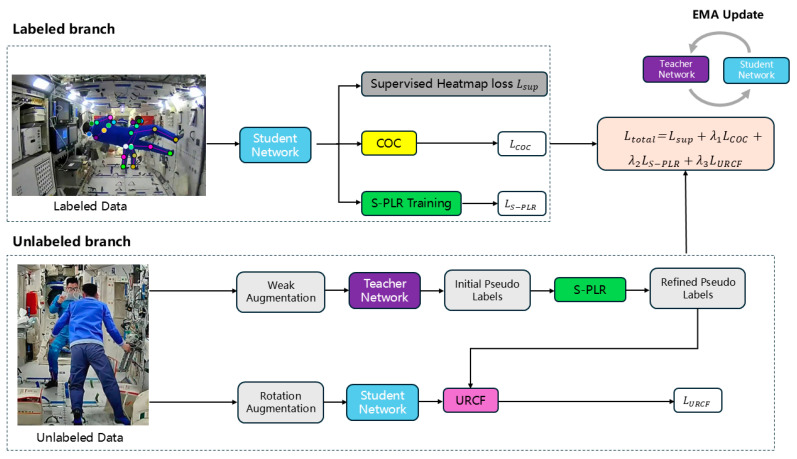
Overall architecture of the proposed physics-inspired semi-supervised human pose estimation framework. The upper branch corresponds to labeled training, where the Student network is optimized not only by the basic supervision but also by COC and the labeled-only training of S-PLR. The lower branch corresponds to unlabeled training, where the Teacher network first generates initial pseudo labels, which are then refined by S-PLR to obtain refined pseudo labels. These refined pseudo labels, together with the Student predictions on rotation-augmented views, jointly form URCF. The overall training process jointly optimizes the Student network, while the Teacher network is updated through EMA.

Although semi-supervised frameworks such as Mean Teacher have achieved promising results on general pose estimation tasks, the initial pseudo labels generated by the teacher network remain susceptible to structural noise and local deviations due to the complexity of pose distributions and the scarcity of annotations [39]. Meanwhile, large-angle rotations and free-floating motions in microgravity scenarios make it difficult for conventional consistency learning to adequately model the stability of predictions under rotational perturbations. To address these issues, we propose a physics-inspired semi-supervised pose estimation framework. Built upon the Mean Teacher architecture and further incorporating the design principles of MultiAugs for multi-view augmentation and consistency learning, the proposed framework performs joint optimization from three aspects: canonical orientation modeling, structural pseudo-label refinement, and uncertainty-guided consistency learning, as shown in Figure 2.

Specifically, the COC is first introduced to establish a unified global orientation reference in the image plane, thereby mitigating the orientation ambiguity caused by the absence of a natural directional prior in microgravity environments. Next, with the aid of the S-PLR, the initial pseudo labels generated by the teacher network are structurally refined through explicit modeling of human skeletal topology, yielding more reliable pseudo-label supervision anchors. Finally, the URCF is proposed to impose consistency constraints on the student network under multi-angle rotated views, while adaptively weighting the unsupervised loss using sample-level uncertainty to suppress confirmation bias introduced by noisy pseudo labels.

Within this framework, the Teacher branch is kept as a pure EMA [40] branch throughout training and is exempt from backpropagation. All additional training objectives are applied only to the Student branch or to the refinement module under labeled supervision, so as to maintain consistency and stability during training. To improve the reliability of pseudo-label generation, the Teacher parameters are not updated by gradient descent, but solely by the exponential moving average of the Student parameters, formulated as follows:

(2) $$\theta_{t}^{\left(k\right)}=\lambda_{\mathrm{ema}}\theta_{t}^{\left(k-1\right)}+\left(1-\lambda_{\mathrm{ema}}\right)\theta_{s}^{\left(k\right)}$$

where $\theta_{t}^{\left(k-1\right)}$ denotes the Teacher parameters from the previous iteration, $\theta_{s}^{\left(k\right)}$ denotes the Student parameters at the current iteration, and $\lambda_{\mathrm{ema}}$ is the EMA momentum coefficient.

### 3.2. Canonical Orientation Constraint

**Figure 3:**
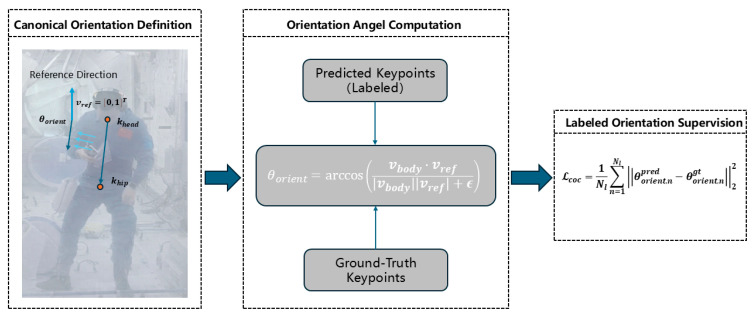
Illustration of the Canonical Orientation Constraint. A unified reference direction is defined in the image plane, and the body-axis orientation is constructed from the head and hip keypoints, with orientation consistency enforced on labeled samples.

Motivated by the orientation-prior mismatch discussed in the Introduction, COC is designed to provide an explicit orientation-level regularization for microgravity pose estimation. Most conventional human pose estimation models are developed on terrestrial scene data and therefore implicitly benefit from the stable orientation distribution induced by gravity during training. In microgravity environments, however, astronauts can freely float and rotate inside the cabin, and the overall body orientation in the image no longer follows the statistical regularities commonly observed in ground-based scenarios. This physical characteristic motivates the use of a microgravity-related orientation prior to the proposed framework, although no explicit force, dynamic, or kinematic model is involved. If the representation bias learned from terrestrial data is directly retained, the model may incorrectly interpret global orientation changes as local structural variations, which in turn leads to unstable keypoint predictions. To alleviate this issue, COC [41] is introduced to establish a unified global orientation reference in the image plane, thereby explicitly constraining the representation of overall human body orientation and reducing pose ambiguity caused by the absence of a natural directional prior in microgravity environments, as illustrated in Figure 3.

It should be emphasized that the proposed orientation constraint does not directly model the actual physical gravity in microgravity environments. Instead, it is inspired by the gravity-induced orientation bias commonly observed in terrestrial scenes and introduces a canonical orientation prior to compensate for the absence of a natural vertical tendency under microgravity conditions. To this end, we define a reference direction vector $v_{\mathrm{ref}}={\left[0,1\right]}^{T}$, corresponding to the vertical direction in the camera pixel coordinate system. Let the human keypoint set be denoted as $K=\{k_{1},k_{2},\ldots ,k_{N}\}$, where $k_{i}=\left(x_{i},y_{i}\right)$. We define the body-axis vector as $v_{\mathrm{body}}=k_{\mathrm{hip}}-k_{\mathrm{head}}$ using the head and hip keypoints. The absolute body orientation angle relative to the environment, denoted by $\theta_{\mathrm{orient}}$, is defined as:

(3) $$\theta_{\mathrm{orient}}=\arccos\left(\frac{v_{\mathrm{body}}\cdot v_{\mathrm{ref}}}{|v_{\mathrm{body}}||v_{\mathrm{ref}}|+\epsilon }\right)$$

where ϵ is a small constant introduced to prevent division by zero in the denominator. Under the above definition, $\theta_{\mathrm{orient}}^{\mathrm{pred}}$ is deterministically computed from the geometric relationships among the predicted keypoints, while for labeled samples, $\theta_{\mathrm{orient}}^{\mathrm{gt}}$ is obtained from the ground-truth keypoint coordinates.

Since unlabeled data do not provide ground-truth orientation annotations, we impose a global orientation constraint only on the labeled dataset $D_{l}$ by enforcing consistency between the predicted orientation angle $\theta^{pred}$ and the ground-truth orientation angle $\theta^{\mathrm{gt}}$:

(4) $$L_{\mathrm{coc}}=\frac{1}{N_{l}}\sum_{n=1}^{N_{l}}{\left|\left|\theta_{\mathrm{orient},n}^{\mathrm{pred}}-\theta_{\mathrm{orient},n}^{\mathrm{gt}}\right|\right|}_{2}^{2}$$

This constraint is applied only to the labeled branch, but it can still improve the overall representation capacity through the shared feature extractor and pose estimation backbone. As a result, it enhances the stability of the pseudo labels generated by the Teacher network on complex microgravity poses, providing a more reliable basis for subsequent structural refinement and consistency learning.

### 3.3. Structure-Aware Pseudo Label Refinement

**Figure 4:**
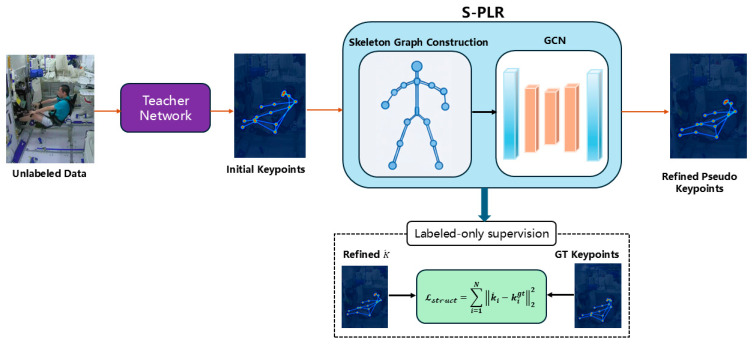
Illustration of the Structure-Aware Pseudo-Label Refinement. The initial keypoint predictions are represented as a human skeleton graph and refined via GCN-based topology propagation. The module is trained only on labeled samples and then used for forward pseudo-label refinement on unlabeled data.

In a semi-supervised framework, the quality of pseudo labels plays a critical role in determining the effectiveness of unlabeled data utilization. In microgravity scenarios, the greater freedom of human body orientations, limited annotated samples, severe occlusion, and motion blur often cause the initial predictions generated by the Teacher network to suffer from local offsets, topological distortions, and structural inconsistencies. If such pseudo labels are directly used to supervise the Student network, these errors may be propagated and accumulated during training, thereby limiting the performance of semi-supervised learning. To address this issue, S-PLR is introduced to structurally refine the initial keypoint predictions generated by the Teacher network before they are used for unsupervised consistency optimization. As depicted in Figure 4, S-PLR explicitly models human skeletal topology and produces more reliable pseudo-label supervision anchors. Rather than focusing on a standalone graph architecture [42], this module is designed as a lightweight structure-aware refinement component within the proposed semi-supervised framework. By introducing skeletal topology modeling, S-PLR constrains the initial pseudo labels at the structural level, thereby improving their plausibility and stability under large rotations, self-occlusion, and cabin background interference.

Specifically, the human skeleton is modeled as a graph *G* = (*V*, *E*), where V denotes the set of (N) keypoints (joints) and E denotes the physical kinematic connections (bones) of the human body. For the initial keypoint predictions generated by the Teacher network, the coordinate set is denoted by $K\in R^{N\times 2}$. This set is then taken as the initial node feature of the graph, denoted by $H^{\left(0\right)}=K\in R^{N\times 2}$. A graph convolutional network (GCN) is then employed to propagate information among keypoints and refine their structural representation. The layer-wise propagation rule is defined as:

(5) $$H^{\left(l+1\right)}=\sigma \left(\tilde{D^{-\frac{1}{2}}}\tilde{A}\tilde{D^{-\frac{1}{2}}}H^{\left(l\right)}W^{\left(l\right)}\right)$$

where $H^{\left(l\right)}$ denotes the hidden node features at the (l)-th layer; $\tilde{A}=A+I$ is the human skeleton adjacency matrix augmented with self-connections, where $A\in \{0,1{\}}^{N\times N}$ defines the physical connectivity between nodes; $\tilde{D}$ is the corresponding diagonal degree matrix, satisfying $\tilde{D_{\mathrm{ii}}}=\sum_{j}\tilde{A_{\mathrm{ij}}}$; $W^{\left(l\right)}$ is the learnable weight matrix of the (l)-th layer; and $\sigma \left(\cdot \right)$ denotes the nonlinear activation function.

After *L* layers of topological information propagation through the GCN, the final refined keypoint coordinate set is denoted as $\hat{K}=H^{\left(L\right)}\in R^{N\times 2}$. To avoid the self-mapping degeneracy caused by using supervision targets derived from the same source as the input on unlabeled samples, the structural refinement module is trained under supervision only on labeled data. Accordingly, the structural constraint loss is defined as the squared Euclidean distance between the refined coordinates and the ground-truth keypoint coordinates:

(6) $$L_{\mathrm{struct}}=\sum_{i=1}^{N}{\left\|\hat{k_{i}}-k_{i}^{\mathrm{gt}}\right\|}_{2}^{2}$$

where $\hat{k_{i}}$ denotes the (i)-th refined keypoint prediction in the final output matrix $\hat{K}$, and $k_{i}^{\mathrm{gt}}$ denotes the corresponding ground-truth keypoint coordinate.

In this module, the structural refinement head of S-PLR is trained only on labeled samples. For unlabeled samples, no additional reconstruction loss is introduced based on Teacher predictions. Instead, the trained structural refinement head is used solely for forward inference-based pseudo-label refinement. This design avoids the identity-mapping issue that may arise when noisy pseudo labels are simultaneously used as both input and target. Although the refinement head is supervised using the labeled subset, it mainly captures relatively stable skeletal topology and local joint dependencies rather than complex image-level appearance representations. These structural relationships are shared between labeled and unlabeled samples, allowing the trained S-PLR module to refine Teacher predictions on unlabeled data without introducing additional manual annotations. Accordingly, the overall structural refinement loss within the S-PLR module can be simplified as:

(7) $$L_{S\text{-}P\mathrm{LR}}=L_{\mathrm{struct}}$$

In the unlabeled branch, the initial keypoint predictions produced by the Teacher network are structurally refined by the trained S-PLR module to obtain more structurally reliable pseudo labels, denoted by $\hat{y_{i}}=\hat{K_{\mathrm{teacher}}}$, which serve as supervision anchors for subsequent consistency learning.

### 3.4. Uncertainty-Guided Rotational Consistency Framework and Overall Optimization

**Figure 5:**
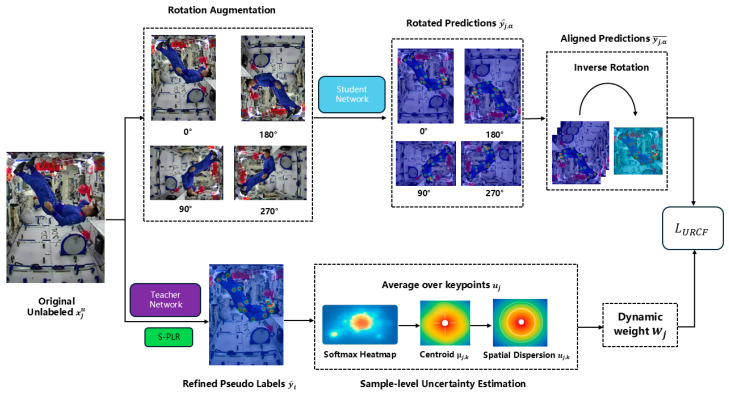
Illustration of the Uncertainty-Guided Rotational Consistency Framework. The Student network predicts on multiple rotated views, and the predictions are inverse-rotated back to the original coordinate frame. The refined pseudo labels are used for both consistency supervision and sample-level uncertainty estimation to generate dynamic weights for the weighted consistency loss.

To further improve robustness under large rotational perturbations in unlabeled samples, URCF introduces uncertainty-aware consistency learning after S-PLR-based pseudo-label refinement. In microgravity scenarios, human poses may appear at arbitrary orientations, making it difficult for conventional consistency constraints alone to adequately capture predictive stability under large-angle rotations and free flipping. Applying unsupervised constraints with the same strength to all samples would allow highly uncertain pseudo labels to introduce additional noise and further exacerbate confirmation bias [43]. To address this issue, we propose the URCF, whose framework is illustrated in Figure 5, to estimate pseudo-label reliability and adaptively weight the rotational consistency loss [44], thereby improving the robustness of the Student network to diverse rotational variations in complex microgravity environments.

Uncertainty Estimation. We first perform sample-level uncertainty estimation on the pseudo labels refined by S-PLR. Let $\{H_{j,k}{\}}_{k=1}^{K}$ denote the keypoint heatmaps of the j-th unlabeled sample, where *K* is the number of keypoints. To characterize the localization uncertainty of the *k*-th keypoint, the spatial dispersion of its heatmap response is adopted as the uncertainty measure. Specifically, the heatmap is first normalized into a spatial probability distribution:

(8) $$P_{j,k}\left(p\right)=\frac{\exp\left(H_{j,k}\left(p\right)\right)}{\sum_{q}\exp\left(H_{j,k}\left(q\right)\right)}$$

where p=(x,y) denotes a pixel location in the heatmap. The heatmap centroid, namely its mathematical expectation, is further defined as:

(9) $$\mu_{j,k}=\sum_{p}P_{j,k}\left(p\right)p$$

Accordingly, the uncertainty of the *k*-th keypoint is defined as the second central moment of this probability distribution, which measures its spatial dispersion:

(10) $$u_{j,k}=\sum_{p}P_{j,k}\left(p\right){\left\|p-\mu_{j,k}\right\|}_{2}^{2}$$

On this basis, the uncertainties of all keypoints are further averaged to obtain a sample-level uncertainty measure:

(11) $$u_{j}=\frac{1}{K}\sum_{k=1}^{K}u_{j,k}$$

The sample-level uncertainty is then mapped to a dynamic confidence weight:

(12) $$w_{j}=\exp\left(-\gamma u_{j}\right)$$

where γ is a scaling coefficient. A larger weight indicates that the current refined pseudo labels are more reliable, whereas a smaller weight suggests that the corresponding sample should be subject to a weaker consistency constraint in subsequent learning.

URCF. After obtaining the sample-level dynamic weights, we further impose a rotational consistency constraint to improve the prediction stability of the Student network under multi-angle rotated views. Let $x_{j}^{u}$ denote an unlabeled sample, and let $y_{j}$ denote its pseudo label refined by S-PLR. For any rotation angle $\left(\alpha \in R\right)$, the prediction of the Student network on the rotation-augmented view is given by:

(13) $$\tilde{y_{j,\alpha }}=f_{\theta_{s}}\left(R_{\alpha }\left(x_{j}^{u}\right)\right)$$

To enable comparison with the pseudo label in the original view, the prediction is further transformed by an inverse rotation and mapped back to the original coordinate system:

(14) $$\bar{y_{j,\alpha }}=R_{\alpha }^{-1}\left(\tilde{y_{j,\alpha }}\right)$$

Accordingly, the unsupervised consistency loss of URCF is defined as:

(15) $$L_{\mathrm{URCF}}=\frac{1}{\left|R\right|N_{u}}\sum_{j=1}^{N_{u}}\sum_{\alpha \in R}w_{j}{\left\|\bar{y_{j,\alpha }}-\hat{y_{j}}\right\|}_{2}^{2}$$

This design enables the model to impose stronger consistency supervision on high-confidence pseudo labels, while automatically reducing the penalty imposed on highly uncertain samples, thereby effectively mitigating the confirmation bias introduced by noisy pseudo labels.

Overall Optimization. For labeled samples, the mean squared error between the predicted heatmaps and the target heatmaps is adopted as the basic supervised loss. Let $P_{s}\left(x_{i}^{l}\right)$ denote the multi-channel keypoint heatmaps produced by the Student network $f_{\theta_{s}}\left(\cdot \right)$ for the input image $x_{i}^{l}$, and let $Y_{i}^{l}$ denote the 2D Gaussian ground-truth heatmaps generated from the annotated keypoint coordinates. The supervised loss is then defined as:

(16) $$L_{\sup}=\frac{1}{N_{l}}\sum_{i=1}^{N_{l}}{\left\|P_{s}\left(x_{i}^{l}\right)-Y_{i}^{l}\right\|}_{2}^{2}$$

By integrating the above components, the overall training objective is formulated as a weighted combination of the supervised loss $L_{sup}$ on labeled data and the regularization terms induced by the orientation, structural, and consistency constraints:

(17) $$L_{\mathrm{total}}=L_{\sup}+\lambda_{1}L_{\mathrm{COC}}+\lambda_{2}L_{S\text{-}P\mathrm{LR}}+\lambda_{3}L_{\mathrm{URCF}}$$

where $\lambda_{1}$, $\lambda_{2}$ and $\lambda_{3}$ are fixed hyperparameters used to balance the relative contributions of different loss terms. In this study, they are set to $\lambda_{1}=0.05$, $\lambda_{2}=0.1$, and $\lambda_{3}=1.0$, corresponding to the COC loss, S-PLR loss, and URCF consistency loss, respectively. The URCF term is assigned a unit weight because it is a heatmap-level consistency loss with a numerical scale comparable to the supervised heatmap loss. By contrast, COC and S-PLR serve as auxiliary regularization terms and are therefore assigned smaller weights to prevent them from dominating the primary keypoint localization objective. These coefficients are kept unchanged across all experiments without dataset-specific re-tuning. Through this jointly optimized multi-constraint objective, the model achieves improved representation stability under complex pose variations in microgravity environments.

## 4. Experiments

To systematically validate the effectiveness of the proposed method for semi-supervised human pose estimation in microgravity environments, experiments are conducted on the public COCO dataset and the self-collected Astro-Pose dataset. The experiments are mainly designed to address the following four questions:

(1) whether the proposed method is competitive on standard benchmarks;
(2) whether it can deliver more substantial performance gains in the target microgravity scenario;
(3) whether each core module remains effective under limited annotation settings;
(4) whether the proposed method can improve keypoint prediction quality on challenging samples.

### 4.1. Experimental Setup

(1) Datasets

COCO. Experiments are first conducted on the COCO human keypoint detection benchmark. As a standard benchmark dataset and keypoint annotation protocol for 2D human pose estimation, COCO is used to evaluate the generalization capability of the proposed framework in natural scenes [20]. Following common semi-supervised settings, 1K, 5K, and 10K labeled samples are adopted, while the remaining training images are treated as unlabeled data.

**Figure 6:**
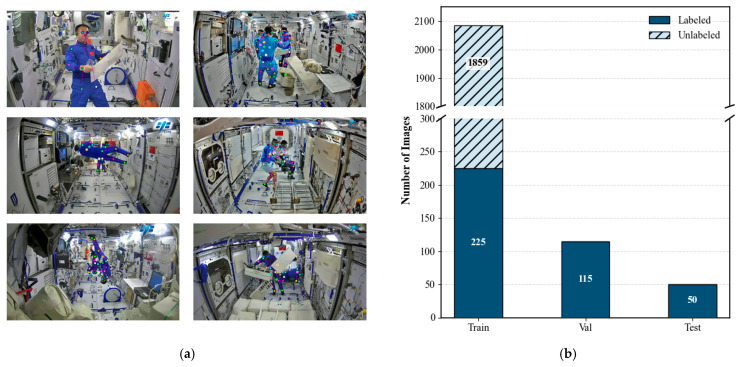
Astro-Pose dataset overview. (**a**) Representative microgravity samples. (**b**) Dataset split statistics.

Astro-Pose. To evaluate the practical effectiveness of the proposed method in the target microgravity scenario, we construct a dedicated in-orbit astronaut pose image dataset, termed Astro-Pose, as shown in Figure 6. The dataset is collected from publicly available video clips recorded aboard space stations. Frames containing clearly visible human subjects are manually sampled, yielding a total of 2249 images, and annotated using the CVAT tool in the COCO-style keypoint annotation format. This design keeps the annotation format consistent with COCO, thereby enabling unified training, evaluation, and comparison across the public and target-domain datasets. Following the COCO annotation protocol, each keypoint is assigned a visibility flag during labeling. Visible keypoints are directly annotated, partially occluded but inferable keypoints are marked as occluded, and unreliable keypoints caused by severe occlusion, truncation, or motion blur are marked as unlabeled and excluded from the corresponding supervised loss. After annotation, all labeled samples are manually reviewed to reduce localization errors, incorrect visibility assignments, missing joints, and skeleton-topology inconsistencies. Specifically, Astro-Pose consists of 225 labeled training images, 1859 unlabeled training images, 115 validation images, and 50 test images. The labeled subset accounts for approximately 10.8% of the training data, while the remaining training images are used as unlabeled samples, forming a typical semi-supervised setting with limited annotations and abundant unlabeled data. Compared with ground-based natural scene datasets, Astro-Pose contains a large number of challenging samples with inverted floating poses, large-angle rotations, self-occlusion, and occlusion caused by cabin equipment, making it more representative of the real challenges encountered in space-station visual monitoring tasks.

(2) Evaluation Metrics

The COCO-style evaluation metrics, including AP, AP50, AP75, and AR, are adopted in this study. Among them, AP measures the overall keypoint localization accuracy, AP50 reflects detection performance under a relatively loose threshold, AP75 places greater emphasis on fine-grained localization under stricter criteria, and AR is used to evaluate the overall recall capability of the model. For microgravity datasets such as Astro-Pose, which exhibit arbitrary pose orientations and complex occlusions, AP75 and AR are more indicative of the model’s true performance in terms of structural stability and precise localization.

(3) Implementation Details

All experiments are implemented under a top-down 2D human pose estimation framework, with ResNet-18 and ResNet-50 adopted as the backbone networks. These two backbones are selected to provide stable and reproducible baselines with different model capacities: ResNet-18 represents a relatively lightweight setting, whereas ResNet-50 offers stronger representation capability. Compared with MediaPipe, PoseNet, and YOLO-based pose estimators, which mainly focus on real-time general-purpose inference, this framework is more suitable for controlled analysis of the proposed semi-supervised modules in microgravity scenes. In semi-supervised training, the overall framework follows the Mean Teacher paradigm, where the teacher network provides stable pseudo-supervision signals for the student network, and the teacher parameters are updated as the exponential moving average of the student parameters. Following the implementation protocols of existing semi-supervised pose estimation methods, the input image resolution is set to 256 × 192. Training is conducted on two RTX 3090 GPUs with a batch size of 32, using the Adam optimizer with an initial learning rate of 1 × 10^−3^. The learning rate is decayed to 1 × 10^−4^ and 1 × 10^−5^ at the 70th and 90th epochs, respectively, and the model is trained for a total of 100 epochs. It should be noted that the proposed COC, S-PLR, and URCF components are mainly used during offline semi-supervised training to improve pose-estimation robustness under limited annotations. During inference, the teacher network and auxiliary pseudo-label optimization procedures are removed, and only the trained student pose estimator is retained. Therefore, the proposed framework does not introduce additional inference-time teacher branches or pseudo-label refinement procedures compared with the baseline pose estimator. The inference-time model size and computational cost are mainly determined by the selected pose-estimation backbone and head, rather than by the auxiliary semi-supervised training components.

### 4.2. Comparison with State-of-the-Art Methods

(1) Quantitative Analysis

We first compare the proposed method with several representative semi-supervised human pose estimation approaches on the COCO dataset, including PseudoPose [25], PoseCons [25], SSPCM [26], and MultiAugs [27]. Table 2 presents the results obtained with ResNet-18, where the proposed method achieves 44.2 AP, 55.7 AP, and 58.8 AP under the 1K, 5K, and 10K labeled settings, respectively. Table 3 reports the corresponding results with ResNet-50, under which the performance further improves to 48.2 AP, 60.5 AP, and 64.6 AP, respectively. These results show that, although some metrics on COCO are slightly lower than those of the state-of-the-art semi-supervised baseline MultiAugs (Dual), the proposed method still maintains competitive performance compared with other general-purpose methods developed for ground-based scenes. This is reasonable because COC and URCF are specifically designed to address omnidirectional pose distributions and large rotational perturbations in microgravity. When applied to conventional scenes such as COCO, these constraints may introduce a certain degree of over-regularization, leading to a modest reduction in accuracy. Therefore, the COCO experiments mainly serve to verify the general competitiveness of the proposed framework on a standard benchmark, while the Astro-Pose experiments provide a more direct evaluation of its effectiveness in the target microgravity scenario.

**Figure 7:**
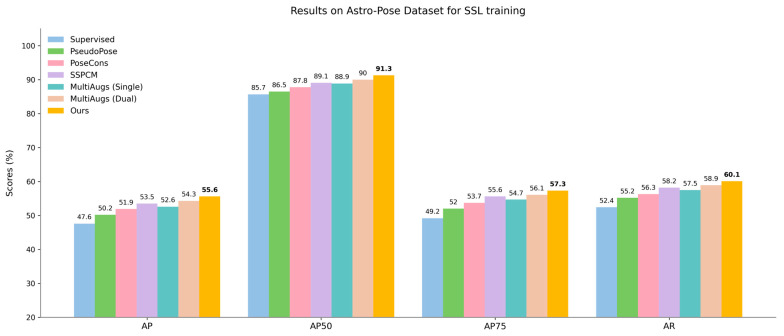
Comparison of different methods on the Astro-Pose dataset. Bold values indicate the best results.

We further compare all methods on the self-constructed Astro-Pose dataset. Table 4 summarizes the quantitative results, from which it can be observed that the purely supervised baseline achieves only 47.6 AP, 85.7 AP50, 49.2 AP75, and 52.4 AR, whereas existing semi-supervised methods all improve upon this baseline to varying degrees. The overall performance comparison is further illustrated in Figure 7. Among these methods, the proposed approach delivers the best overall performance, achieving 55.6 AP, 91.3 AP50, 57.3 AP75, and 60.1 AR. Relative to the supervised baseline, it improves AP by 8.0 points and AP75 by 8.1 points, demonstrating the effectiveness of exploiting unlabeled data. Moreover, the proposed method consistently outperforms the strong baseline MultiAugs (Single), indicating that the observed gains are mainly attributable to targeted modeling of microgravity-specific characteristics rather than simply increased training complexity. The proposed modules operate in a complementary manner. The COC effectively alleviates orientation ambiguity in microgravity, thereby improving the stability of initial feature extraction and pseudo-label generation. The S-PLR module corrects topological distortions under extreme poses and produces highly reliable pseudo-label anchors. The URCF module further suppresses confirmation bias under omnidirectional perturbations, substantially enhancing the model’s generalization ability and robustness to noise under complex pose distributions.

(2) Qualitative Analysis

**Figure 8:**
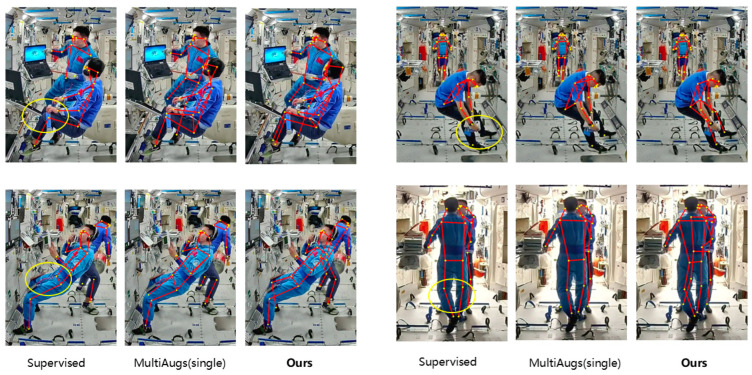
Qualitative comparison of skeleton-based pose estimation results on challenging microgravity samples. Red lines and yellow dots indicate skeleton connections and keypoints, respectively. Yellow circles highlight representative regions with noticeable prediction differences.

To further validate the practical prediction performance of the proposed method in complex microgravity scenarios, we conduct a qualitative visual comparison on the Astro-Pose test set. Figure 8 visualizes the predicted poses as skeleton overlays, enabling a direct comparison of structural consistency and keypoint localization accuracy. The representative examples indicate that existing methods are more prone to limb confusion, keypoint drift, and low-confidence local predictions in challenging cases involving large-angle rotations, inverted poses, self-occlusion, limb overlap, and occlusion caused by cabin equipment or complex background interference.

By contrast, the proposed method produces more stable keypoint predictions that better preserve human topological structure, showing superior structural consistency and localization accuracy on challenging microgravity samples. This observation is consistent with the improvements in AP75 and AR observed in the quantitative results, suggesting that the proposed method not only improves overall detection performance but also enhances localization capability and overall robustness under complex microgravity conditions.

### 4.3. Ablation Study

**Figure 9:**
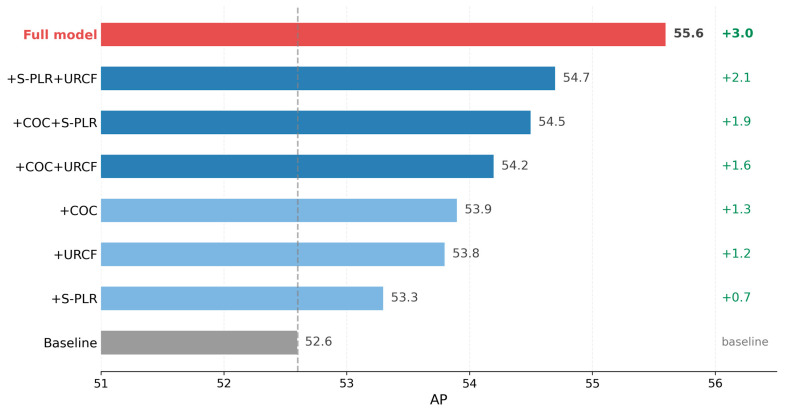
Ablation study on the Astro-Pose dataset.

To analyze the contribution of each component to the overall performance, ablation experiments are conducted on the Astro-Pose dataset using the baseline framework corresponding to MultiAugs (Single) as the reference. As COC, S-PLR, and URCF function as training-time constraints or pseudo-label refinement mechanisms rather than independent inference stages, their effects are assessed through ablation experiments and qualitative comparisons. Table 5 presents the quantitative results, while Figure 9 provides a visual comparison of different ablation settings. The baseline model achieves 52.6 AP. Introducing COC, S-PLR, or URCF individually improves the performance to 53.9 AP, 53.3 AP, and 53.8 AP, respectively, demonstrating that all three components are individually effective. Specifically, COC alleviates orientation ambiguity in microgravity scenarios, S-PLR improves the structural reliability of Teacher-generated pseudo labels by incorporating skeletal topology constraints, and URCF enhances robustness to rotational perturbations through uncertainty-guided consistency learning.

When any two modules are combined, the performance further improves to 54.5 AP, 54.2 AP, and 54.7 AP, respectively, demonstrating strong complementarity among the proposed components. The combination of S-PLR and URCF yields the largest two-module gain, indicating that structurally refined pseudo labels can provide more reliable supervision for uncertainty-guided rotational consistency learning. When all three modules are enabled, the model reaches 55.6 AP, outperforming the baseline by 3.0 AP. These results suggest that orientation regularization, structure-aware pseudo-label refinement, and uncertainty-guided consistency learning jointly contribute to the improvement of semi-supervised pose estimation in microgravity scenarios.

### 4.4. Label Ratio Analysis

**Figure 10:**
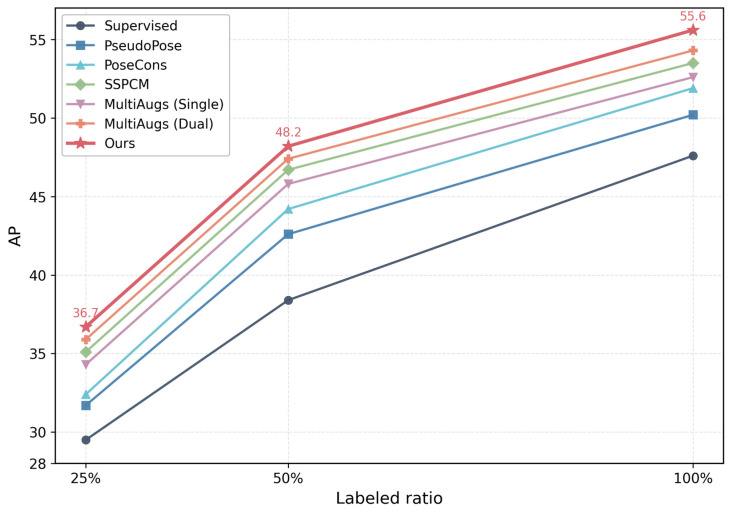
Performance variation under different label ratios on the Astro-Pose dataset.

Given that this study focuses on semi-supervised human pose estimation under limited annotation, evaluating model performance at only a fixed annotation scale is insufficient to fully reflect its capacity to exploit unlabeled data. We therefore further analyze the performance of different methods under varying label ratios on the Astro-Pose dataset. Table 6 summarizes the quantitative results, and the corresponding performance trends are further illustrated in Figure 10. Specifically, when the proportion of labeled data is set to 25%, 50%, and 100%, the proposed method achieves 36.7 AP, 48.2 AP, and 55.6 AP, respectively, consistently outperforming the purely supervised baseline. This observation suggests that the proposed method is more capable of exploiting the latent information contained in unlabeled data when labeled samples are extremely scarce. Further comparison with existing semi-supervised methods shows that the proposed method maintains the best performance across all label ratios. Overall, these results demonstrate that the proposed COC, the S-PLR, and the URCF mechanism can more effectively alleviate orientation ambiguity, pseudo-label noise, and complex pose perturbations in microgravity scenes under limited annotation, making the method well-suited to practical applications such as astronaut pose estimation, where annotated data are inherently scarce.

## 5. Conclusions

This paper presents a physics-inspired semi-supervised human pose estimation framework for microgravity scenarios. To address orientation ambiguity, pseudo-label noise, and severe rotational perturbations, the proposed method incorporates a Canonical Orientation Constraint, a Structure-aware Pseudo-Label Refinement module, and an Uncertainty-Guided Rotational Consistency Framework within a Mean Teacher architecture. By jointly modeling canonical orientation priors, structurally refining pseudo labels, and adaptively weighting rotational consistency learning, the proposed framework improves the robustness and generalization ability of pose estimation under complex microgravity conditions. Extensive experiments on both COCO and the self-constructed Astro-Pose dataset demonstrate its effectiveness. In particular, under limited annotation, the proposed method achieves more accurate and stable pose predictions in challenging microgravity scenes with large rotations and frequent occlusions. Future work will further evaluate the framework on more diverse on-orbit data and incorporate temporal cues from in-orbit videos, with the goal of extending keypoint-level 2D pose estimation toward pose-type or action-category recognition and enabling more fine-grained quantitative analysis of astronaut motion patterns in microgravity.

## Figures and Tables

**Table 1:** Summary of representative pose estimation studies and the positioning of the proposed method.

Category	Representative Methods	Research Focus	Gap Addressed by This Study
General 2D HPE	OpenPose [3], HRNet [13], ViTPose [4]	General human keypoint localization	Limited robustness to arbitrary body orientations in microgravity scenes
Lightweight HPE	PoseNet [14], MediaPipe BlazePose [15], YOLO-pose [16]	Real-time inference and deployment	Limited adaptation to microgravity-specific orientations and cabin occlusions
Semi-supervised HPE	PseudoPose [17], PoseCons [17], SSPCM [18], MultiAugs [19]	Pseudo-labeling and consistency learning with limited labels	Pseudo labels are sensitive to rotation, occlusion, and structural noise
Space-related HPE	μPose [6], AstroPose [20], H^3^-Astronaut Pose Net [5], AstroHSP [21]	Astronaut pose perception in space-related scenes	Existing solutions often rely on 3D multi-view input, synthetic data, or task-specific assumptions
Ours	PhysAstro-Pose	Label-efficient monocular 2D HPE in microgravity scenes	Jointly addresses scarce annotations, orientation ambiguity, structural pseudo-label noise, and rotational uncertainty

**Table 2:** AP of different methods on COCO when different numbers of labels are used. Backbone is ResNet18.

Methods	1K	5K	10K	All
Supervised [27]	31.5	46.4	51.1	67.1
PseudoPose [17]	37.2	50.9	56.0	—
PoseCons [17]	42.1	52.3	57.3	—
SSPCM [18]	46.9	57.5	60.7	—
MultiAugs (Single) [19]	45.5	56.2	59.9	—
MultiAugs (Dual) [19]	**49.7**	**58.8**	**61.8**	—
Ours *	44.2	55.7	58.8	—

* “Ours” denotes the proposed method. Bold values indicate the best performance under each setting.

**Table 3:** AP of different methods on COCO when different numbers of labels are used. Backbone is ResNet50.

Methods	1K	5K	10K	All
Supervised [27]	34.8	50.5	56.4	70.9
PoseCons [17]	43.1	57.3	61.8	—
SSPCM [18]	49.7	61.8	65.4	—
MultiAugs (Single) [19]	49.4	61.4	65.3	—
MultiAugs (Dual) [19]	**51.6**	**62.9**	**66.3**	—
Ours *	48.2	60.5	64.6	—

* “Ours” denotes the proposed method. Bold values indicate the best performance under each setting.

**Table 4:** Results on Astro-Pose Dataset for SSL training. Backbone is ResNet18.

Methods	AP	AP50	AP75	AR
Supervised [27]	47.6	85.7	49.2	52.4
PseudoPose [17]	50.2	86.5	52.0	55.2
PoseCons [17]	51.9	87.8	53.7	56.3
SSPCM [18]	53.5	89.1	55.6	58.2
MultiAugs (Single) [19]	52.6	88.9	54.7	57.5
MultiAugs (Dual) [19]	54.3	90.0	56.1	58.9
Ours *	**55.6**	**91.3**	**57.3**	**60.1**

* “Ours” denotes the proposed method. Bold values indicate the best performance under each setting.

**Table 5:** Ablation study of the proposed modules on the Astro-Pose Dataset. Backbone is ResNet18.

Baseline	COC	S-PLR	URCF	AP
√				52.6
√	√			53.9
√		√		53.3
√			√	53.8
√	√	√		54.5
√	√		√	54.2
√		√	√	54.7
√	√	√	√	**55.6** *

* Bold values indicate the best performance under each setting.

**Table 6:** AP of different labeled data ratios on the Astro-Pose Dataset. Backbone is ResNet18.

Labeled Ratio	25%	50%	100%
Supervised [27]	29.5	38.4	47.6
PseudoPose [17]	31.7	42.6	50.2
PoseCons [17]	32.4	44.2	51.9
SSPCM [18]	35.1	46.7	53.5
MultiAugs (Single) [19]	34.3	45.8	52.6
MultiAugs (Dual) [19]	35.9	47.4	54.3
Ours *	**36.7**	**48.2**	**55.6**

* “Ours” denotes the proposed method. Bold values indicate the best performance under each setting.

## Data Availability

The datasets presented in this article are not readily available because they are subject to confidentiality requirements and are part of an ongoing study. Requests to access the datasets should be directed to the corresponding author.
